# Characteristics of convicted male-on-female rapists in the South of Sweden between 2013 and 2018: a pilot study

**DOI:** 10.1080/20961790.2020.1757245

**Published:** 2020-05-25

**Authors:** Emelie Stiernströmer, Marie Väfors Fritz, Caroline Mellgren, Ardavan Khoshnood

**Affiliations:** a Department of Criminology, Malmö University, Malmö, Sweden;; b Department of Clinical Sciences Lund, Skane University Hospital, Emergency Medicine, Lund University, Lund, Sweden

**Keywords:** Forensic sciences, crime characteristics, frequency analysis, offender characteristics, rape, victim characteristics

## Abstract

The objective of this study was to evaluate the main characteristics of adult male offenders convicted of rape or aggravated rape against adult females. We reviewed all convictions (*n* = 21) based on court documents from the District Court, the Court of Appeal and information from the Swedish Tax Agency in Malmö, Sweden, between 2013 and 2018. The findings indicated that the most common offender characteristics were a single status, mean age of 33 years and foreign background. The rapes primarily occurred within a private setting while the victims (who were often younger and knew the offender) were unconscious. Although assumptions based on these results should be made with caution, our findings provide a clear image of the typical circumstances under which these rapes occurred. This study should be viewed as a first attempt to create a database of characteristics of convicted rapists. As more data are added, more sophisticated analyses can be performed and stronger generalizations may be made. Information of this kind may also be important for further research, classification of rapists in offender profiling, and case linking.

## Introduction

Recent statistics show that violent crime, including rape, occurs with disturbing frequency in Western societies, and Sweden is no exception [1–4]. However, it is concerning that current understanding of the main characteristics of convicted Swedish rapists, their crimes and their victims is poor. In UK, USA and Canada, this type of scientific knowledge has been used to guide clinical practice; for example, in risk assessments and therapeutic interventions [5]. Such information may also be used for research purposes. In addition, when a large body of data has been compiled, it may also be used in criminal investigations by the police. Despite public concern and increased awareness about rape, the topic of rape and its characteristics in modern-day Sweden has attracted limited scientific interest, and little research on Swedish rapes has been conducted. Although some recent studies that used Swedish samples have made important contributions to the literature [6–9], the current understanding of rape is primarily derived from studies and literature from English-speaking countries. To understand whether this knowledge about rape characteristics is generalizable to countries with different socio-cultural contexts, more rape research must be conducted in Sweden. The objective of this study was to characterize the distribution of the main features of all adult male-on-female convictions that occurred in the city of Malmö, in the south of Sweden, between 2013 and 2018.

### Definition of rape and rape statistics in Sweden

Rape, defined and discussed in Chapter 6 of the Brottsbalken (Swedish Penal Code) [10], states that a rapist can be a man or a woman and rape can be committed against a man, woman or child. Furthermore, the current definition of rape in Sweden does not require that the rape involves penile-vaginal penetration (as it can be defined as penetration by objects or fingers). According to the law, a case is deemed to be rape if it is judged by a court to be of a similar insulting nature to penile penetration, and the overall circumstances are comparable with intercourse.

Statistics from the Brottsförebyggande rådet, acronymed BRÅ (Swedish National Council for Crime Prevention) show that the number of reported rapes in Sweden increased by 34% between 2008 and 2016. In addition, there was a 10% increase in reported rapes from 2016 to 2017 [4, 11]. In 2018, a total of 22 500 sex offences were reported, representing a 2% increase from 2017, and the number of rapes reported to police increased by 8% (to 7 960) between 2017 and 2018. Overall, the number of reported rape offences has increased over the last decade (2009–2018). In 2018, 7 920 rape offences were processed in Sweden, which was similar to the previous year. Of these, an investigation was commenced for 95% of cases, and 5% were dismissed with no investigation. The conviction rate in 2018 was 17%, compared with 31% in 2009 [12]; therefore, there is an emerging trend of increasing reported rapes and decreasing rape convictions. What causes the escalation in rape reports remains unknown, as it cannot be determined whether rapes occurred more frequently or if they were reported more often. However, this trend should be seen as alarming and a sign that empirical knowledge of rapes in Sweden must be improved. One way this could be achieved is studying rapes that had been committed in which the criminal proceedings led to a successful rape conviction. This is because convictions provide the most information, and allow both police reports and court documents to be inspected. Although this approach prevents the outcome being generalized to all rapes (e.g. unreported rapes or those without criminal proceedings) and includes the risk that the convictions are tainted by rape myth and bias in court case progression decisions [13], it offers a clear image of the circumstances under which rape occurs.

Most of the information about rape in Sweden is provided by BRÅ, a knowledge centre for the Swedish criminal justice system. Regrettably, this information does not sufficiently increase empirical knowledge about rape, as its reports on sex crimes are primarily based on police reports or phone surveys. Second, rape convictions are given little attention in its reports. Third, many informative variables that would improve the knowledge of rape are not included in BRÅ reports (e.g. characteristics of the offender are not prioritised). Fourth, when rape characteristics are compared across Sweden’s three large cities (Stockholm, Gothenburg and Malmö), there is no differentiation between adult rape and rape against a child [14]. However, the occasional comparisons of crime statistics have shown that Malmö has a higher number of reported violent crimes in general [14, 15]. Importantly, Malmö has the second-highest number of reported sexual crimes (after Stockholm) based on crime rates per 100 000 citizens. This may partly be attributable to Malmö’s geographical location, population movement patterns or the socioeconomic situation in this particular city. The average number of annual rapes committed by an adult male on an adult female (older than 18 years) reported to the Malmö police was 96.6 (total of 483 reported crimes) between January 2014 and March 2018. The average number of annual criminal rape charges, including attempted rape, in Malmö, brought forward to the city’s District Court by the prosecutor was 11.67 (total of 70 cases between January 2013 and March 2018). Between 2013 and 2018, the Hovrätten över Skåne och Blekinge (the Scania and Blekinge Court of Appeal, in Swedish) convicted 21 adult males for rape/aggravated rape of adult women in Malmö. What constitutes the primary rape characteristics of these recent convictions is currently unknown and was, therefore, the focus of this study. English-speaking countries have come considerably further in an attempt to study different features of rape.

### Characteristics of offenders, crimes and victims

A review of the rape literature indicated that frequently studied components of offenders’ sociodemographic variables included their educational background, employment, ethnicity, age at the time of the offence and civil status [5, 16, 17]. This research suggested that rapists tend to be characterized by poor education, and employment in unskilled professions [18, 19]. However, little is known about ethnicity, which is a more controversial factor when characterizing rapists [20]. Wright and West [21] concluded that rapes conducted in England were predominantly intra-racial, with an over-representation of ethnic minorities as both victims and offenders. However, Biljeveld and Hendricks [22] did not support those results. Because of the limited research on the ethnicity of rapists, it is not yet possible to conclude whether variations in ethnicity are a function of the country from which the data were collected, the sampling techniques used or definitions of ethnicity. An important factor to consider is the national context of the society in which the data were collected (e.g. history of migration, ethnicity and multiculturalism). Regarding offender age, although offenders tend to be older than their victims, rape, like most crimes, is often committed by young males [18, 23, 24]. Various features of rape are dynamic but also influence one another. Previous studies have analysed offenders’ overt crime scene behaviour to provide empirical research regarding offender heterogeneity [25]. The underlying structure of sex crimes (i.e. their different behavioural themes) based on such research has been used to differentiate offences and offenders [6, 26]. Rape can also be the result of an interaction between the offender and the victim within a situational context. It is, therefore, useful to study other crime scene details, such as the victim’s behaviour, and if the offender or victim had been drinking alcohol before the assault [19, 27–29]. Studies on rape characteristics have considered the manner of penetration, offender-victim relationship [30], when and where the rape occurred [31] and victim resistance [32]. Age and civil status are also well-established correlates of criminal victimization, including rape [33], which is a crime primarily perpetrated against younger women [6, 19].

### The present study

As evident from reviewing the rape literature, much can be learned about rape from examining the characteristics of the offenders, crimes and victims in rape cases. This can be achieved in different ways. For example, using different sources of information (e.g. court documents, police reports and interviews) as well as considering different variables. The focus of the present study was to portray the main characteristics (frequency and percentage) of all convicted adult male-on-female rapes that occurred between 2013 and 2018 in Malmö.

## Methods

### Material

All cases in which male offenders were convicted of rape against adult females between 2013 and 2018 in Malmö were included in this study. The restriction to adult male-on-female rapes was partly because few convicted female rapists were to be expected as females are more often victimized [12]. Another reason was the limited existing knowledge of female sex offenders, and the significant differences between male and female sexual offenders [34]. Of the reviewed cases, one offender and one victim were aged below 18 years at the time of the crime. However, as the offender was above 18 years and the victim was 17.9 years when the conviction was entered, both cases were included in the sample.

An opt-out model of informed consent was used, where data were collected and used automatically unless the offender actively dissented following information concerning the study posted on the university website. No one withdrew from this study. A total of 24 rape cases in which the conviction included rape or aggravated rape were collected from the Malmö Tingsrätt (the District Court in Malmö, in Swedish) (i.e. the total number of cases in which an adult male was convicted of rape between 2013 and 2018). Twenty-one of these cases had appealed to the Scania and Blekinge Court of Appeal. The conviction was fully overturned in two cases and the appeal was ongoing in one case. These three cases were therefore excluded from the analyses, leaving a total of 21 convictions. None of the final convictions included multiple offenders or multiple victims; however, in two cases, the convicted offender had an accomplice during the crime but the accomplice was not convicted of rape. The offenders were convicted of rape in 18 of the 21 cases. The three remaining offenders were convicted of aggravated rape.

The use of court documents is a common and validated method in criminological research [35]. In the present study, all available court file documents from the District Court of Malmö and the Scania and Blekinge Court of Appeal were reviewed (i.e. verdict, police investigation and forensic investigation). Because of inconsistencies in how offenders’ ethnicity was reported in the court documents, this information was obtained from the Swedish Tax Agency. The study was approved by the local regional ethics review board (Dnr 2018/160).

### Variables

The characteristics concerned convicted offenders, the crimes and the victims. These were coded numerically according to the number of categories for each variable; for example, age was divided into four categories and coded as “1” for offenders aged 18–19 years, “2” for offenders aged 20–30 years, and so on.

The characteristics of the offenders included four variables: conviction age (18–19 years, 20–29 years, 30–39 years, ≥40 years), ethnicity (foreign, Swedish, no record), civil status (single, with a partner), and employment (employed, unemployed, no record). The date of birth and sentence date were used to calculate the conviction age of the offender (as well as the victim). In accordance with the BRÅ definition, an offender’s ethnicity was classified as “foreign” if they had at least one parent from a foreign country or was born abroad [36]. In other cases, ethnicity was defined as Swedish. Civil status contained two categories: “single” (which included widowed and divorced offenders at the time of the crime) and “in relationship”, which included couples and married offenders (the same definition was used for the victim’s civil status). Concerning employment, the “unemployed” category included offenders that were not employed in the traditional sense (e.g. an internship or had daily activities) or when records indicated that they had recently lost a job without further specification.

The crime characteristics contained nine variables. These were target penetration (vaginal, anal, oral, mix, other), offender–victim relationship (stranger, acquaintance, ex-partner, partner), verbal threats towards the victim (no threats, death threats, other threats), crime scene (public outside, offender’s home, victim’s home, other), victim’s state of consciousness (conscious, unconscious), victim’s resistance during the rape (no resistance, resistance, no record), the month of rape (December–February, March–May, June–August, September–November), weekday of rape (Friday–Sunday, Monday–Thursday, no record) and time of rape (noon–6 pm, 6 pm–midnight, midnight–6 am).

Target penetration was defined as completed penetration and the categories used were decided based on the type or combinations of penetrations as stated in the studied documents. Penetration using objects or fingers (without penile-vaginal or anal penetration) was classified as “other”, whereas “mix” indicated that the offender penetrated the victim with his genitals in one of two ways (i.e. vaginal/anal or vaginal/oral). The offender–victim relationship was defined as a “stranger” if the rapist did not know the victim or had known the victim only for a brief period of time (e.g. met at a bar a few hours before the assault). “Acquaintance” was defined as when the victim and the offender had personal knowledge of each other (a person they knew slightly, but who was not a close friend or partner). The location of the crime (i.e. crime scene) was defined as “public outside” in cases where the rape occurred in the outdoors on “public property” such as outside a bar or an area with greenery. Crime scenes that were not in the private realm of the offender or the victim, nor a property dedicated to public use (e.g. taxi, hotel room, or hospital bed) were defined as “other”.

The victim characteristics had three variables: age (18–19 years, 20–29 years, 30–39 years, ≥40 years), alcohol use during the crime (yes, no, no record) and civil status (single, in relationship).

### Procedure

Following contact with the District Court, a list of convicted rapists was created and relevant court documents compiled dating back 5 years. This time period was chosen based on the cycle of operation of the country’s District Courts, meaning that all material held by the courts can be destroyed after 5 years, including pre-trial material. In addition, the District Court computer system registry can only search back for 5 years, and cases are no longer searchable through the registry beyond this period [37]. Each case was reviewed and checked against the Court of Appeal records to see if the decision by the District Court was appealed by the defendant or the prosecutor. After coordination of data from the District Court, the Court of Appeal and the Swedish Tax Agency, 21 cases were identified where at least one offender was convicted of rape or aggravated rape.

### Data treatment and statistical analysis

Once the data were gathered, a systematic approach using document analysis was conducted [38]. This included principles of de-contextualization [39] in which relevant parts of the documents were removed from the original context and studied in combination with text related to the same phenomena. This allowed us to study patterns (including individual patterns) as a phenomenon using a matrix of similar characteristics [38–41]. The text content was then coded numerically and exported to SPSS version 24 (IBM Corp., Armonk, NY, USA). Consistent with previous studies, variables with over 50% of missing values were excluded from the analysis [31, 42]. In the present study, two variables exceeded this limit: the offender’s education level and whether excessive force was used towards the victim. Frequency analyses were conducted to determine the distribution of characteristics.

## Results

Tables 1–3 presents the frequency distributions and percentages of the characteristics of the offenders, victims and crimes respectively based on the 21 rape convictions in Malmö between 2013 and 2018. The average age of offenders at the time of sentencing was 32.9 years (standard deviation (SD) = 9.5 years, range 18–55 years). The average age of the victims at the time of sentencing was 28.5 years (SD = 12.5 years, range 18–59 years). The average age difference between offenders and victims at the time of sentencing was 4.3 years (SD = 11.7 years).

**Table 1.** Frequency distribution of the main characteristics of the offender of convicted male-on-female rapists in Malmö, Sweden, 2013–2018 (*n* = 21).

Characteristics	Frequency (*n*, %)
Age (years)	
18–19	2 (9%)
20–29	6 (29%)
30–39	8 (38%)
≥40	5 (24%)
Ethnicity	
Foreign	15 (71%)
Swedish	5 (24%)
No record	1 (5%)
Civil status	
Single	17 (81%)
With partner	4 (19%)
Employment	
Employed	5 (24%)
Unemployed	2 (9%)
Other	8 (38%)
No record	6 (29%)

Note: offender age calculated based on date of birth and date of sentence.

**Table 2.** Frequency distribution of the main characteristics of the victims of convicted male-on-female rapists in Malmö, Sweden, 2013–2018 (*n* = 21).

Characteristics	Frequency (*n*, %)
Age (years)	
18–19	5 (24%)
20–29	9 (42%)
30–39	1 (5%)
≥40	5 (24%)
No record	1 (5%)
Alcohol	
Yes	11 (53%)
No	3 (14%)
No record	7 (33%)
Civil status	
Single	8 (38%)
With partner	13 (62%)

Note: victim age calculated based on date of birth and date of sentence.

**Table 3.** Frequency distribution of the main characteristics of the crimes of convicted male-on-female rapists in Malmö, Sweden, 2013–2018 (*n* = 21).

Characteristics	Frequency (*n*, %)^a^	Characteristics	Frequency (*n*, %)^a^
Target penetration		Victim resistance	
Vaginal	10 (48%)	No resistance	7 (33%)
Anal	1 (5%)	Resistance	10 (48%)
Oral	2 (9%)	No record	4 (19%)
Mix	6 (29%)		
Other	2 (9%)		
Offender–victim relationship		Month of rape	
Stranger	8 (38%)	Dec–Feb	7 (33%)
Acquaintance	7 (33%)	March–May	5 (24%)
Ex-partners	5 (24%)	June–Aug	4 (19%)
Partners	1 (5%)	Sep–Nov	5 (24%)
Verbal threats		Weekday of rape	
No threats	13 (62%)	Friday–Sun	13 (62%)
Death threats	6 (29%)	Mon–Thurs	7 (33%)
Other threats	2 (9%)	No record	1 (5%)
Crime scene		Time of rape	
Public outside	2 (9%)	Noon–6 pm	3 (14%)
Private (offender)	7 (33%)	6 pm–midnight	6 (29%)
Private (victim)	5 (24%)	Midnight–6 am	12 (57%)
Other	7 (33%)		
Victim state of consciousness			
Conscious	6 (29%)		
Unconscious	15 (71%)		

^a^The percentages may not total 100 due to rounding.

## Discussion

The aim of this study was to illustrate the distributions of main characteristics concerning all convicted rapists, their victims and their crimes that occurred in Malmö between 2013 and 2018. We found that our offenders had a mean age of 28.5 years when the crime was committed, and most were foreigners and single. The main target penetration was vaginal. The majority of victims did not know their offenders and had used alcohol before the rape. When compared with other rape studies, we found that some of the main offender and crime characteristics in our sample supported previous findings. The mean age of our sample was similar to a study by Corovic et al. [6] that reported a mean age of 32.1 years. In addition, the penetration style (predominately vaginal intercourse) was similar to that reported in other studies [6, 19]. Other offender characteristics (civil status and employment rate) differed as the present sample of offenders were predominately single (81%) and had a low employment rate (24%) compared with that in the study by Corovic et al. who showed a far lower rate of single offenders (33%) and a higher rate of employment rate (45.5%) [6]. Concerning employment rate, Miller [19] indicated that unstable employment history and low educational level were considered general risk factors related to characteristics of rapists [19]. Another risk factor noted by Miller was non-European ethnicity, which was consistent with the present study as a majority of offenders had a foreign ethnicity.

Speculations for our findings may involve sampling rapists from differing geographical areas with different socioeconomic situations, varying definitions, the inclusion of only certain rapes (e.g. stranger rapes) or mixed rape types (single *vs.* serial rapes) or due to social and legal changes over time. For example, although stranger rapes constituted a minority of the cases in the present study, the rate was still relatively high (38%). It is possible that greater support from the victim for prosecution in these cases had an impact on the conviction rate [13]. In addition, the findings shown in the present study, such as victim relationship and crime location, differed from those recently presented by [11]. However, it is important to note that BRÅ focuses on percentages of the total number of reported sex crimes (from the entire country) and not rape convictions *per se*. Bias in cases that proceeded to court (or those that did not) could also explain the differences between the BRÅ data and our findings.

Our data also showed that more than half of the victims stated that they were not threatened by the offender, which may be partly explained by the majority of victims being unconscious when the offender initiated the rape; therefore, no threats were needed. Regarding the time of the rape, our results were consistent with Scandinavian studies [43], as the rapes in Malmö primarily occurred between December and February, Friday and Sunday, and between midnight and morning. This is consistent with the routine activity theory [44], which emphasizes the environmental opportunity for a motivated offender to cross paths with a suitable target in the absence of an effective guardian or guardianship. The high rate of rape incidence on Fridays to Sundays (midnight to morning) could be partly explained by these days marking a departure from weekly routines.

Concerning the rape victims, our results showed that these women were primarily aged 20–30 years (with a mean age of 28.5 years). Therefore, they were slightly older than the victims reported in other studies using Swedish samples [6]. Moreover, as might be expected for women in this age group, they were more often in a relationship or married. Approximately half of all the victims further stated that they had consumed alcohol around the time the crime was committed. The implication of alcohol in many rapes may be explained by the association between alcohol and socializing [19, 45]. In the present study, the alcohol consumption may in part explain the high percentage of victims (on their own account) who reported that they were not conscious when the crime was initiated, and may also explain the time when most rapes were committed (i.e. midnight to 6 am).

## Limitations

There are important limitations that should be considered when interpreting these findings. First, this study only included convicted rapists. However, focusing on convicted rapists is reasonable when studying the offender, crime and victim characteristics, as much of this information may be unknown in cases that were reported to the police but did not lead to a conviction. However, a risk remains that cases leading to a conviction differed in offender and crime characteristics from cases that did not. For example, there may be selection effects concerning the rapists identified in this study; among the 21 rapists studied (among all the offenders in the 483 rapes reported to the Malmö Police between 2014 and 2018), there may be an overrepresentation of young, inexperienced offenders who were not forensically aware (as compared with the group that were not convicted and therefore not identified in the present study). The effects of rape myths and bias towards the complainant in case progression decisions on the final sample used in the study may also be important factors to consider.

There were also limitations regarding the variables. Because age was only measured at conviction, the exact age at the time of the crime *per se* remains uncertain. However, the majority of rapes occurred within 1 year before the conviction date. Other variables were broadly defined, which may be considered unsatisfactory for various reasons. For example, an offender was classified as a foreigner if he was born outside of Sweden. Our definition of ethnicity, therefore, explains little about the origins of the majority of offenders. Nonetheless, this was the definition granted to us by the ethics committee, and has previously been used by other government agencies [36]. In addition, the ethnicity of the victims was not included. Therefore, our results cannot add any information on whether the majority of rapes in Malmö were racially motivated [21]. Moreover, offenders’ alcohol consumption was not included as a variable, as they were questioned or examined at a later date and available data did not reflect possible alcohol intake around the time of the rape.

The missing data and the small sample size should also be considered limitations, as they could affect assumptions based on these results. The small sample size may be particularly challenging given the heterogeneity that exists between rapists. However, the small sample size is common in criminal research for practical reasons, especially when focusing on rapes that occurred in a limited geographical location and within a limited time frame, as in the present study.

## Conclusions and future directions

Notwithstanding these limitations, this study makes a novel contribution to the rape literature and rape research in Sweden by being the first to examine and categorize the main characteristics of recently convicted rapes that occurred in the south of Sweden. The fact that there is currently no recent Swedish rape study that can be used as a comparison highlights the need for more studies to be conducted in this area.

It should be emphasized that this study should be considered preliminary at best, and preferably viewed as a first attempt to construct a rape database in which new information on characteristics should be added every 5 years. In time as a larger sample size becomes available, more sophisticated and informative analyses can be conducted, and stronger generalizations may be made. This may allow us to track changes in rape patterns over time, reflecting the characteristics that remain stable and those that change. This information could be used for research purposes and may also help identify different clusters of offenders that can be used in offender profiling [46] and case linking [47].

Future studies should attempt to replicate this study to determine whether the findings are comparable to other Swedish cities or between Scandinavian cities. In addition, because of the over-representation of offenders with foreign ethnicity in our sample, further studies could use other definitions that describe offenders’ origins in a more detailed manner. They should also attempt to understand the possible significance of potentially differing sociocultural factors (e.g. views and values related to gender and sexuality). Specifically, it may be worth looking at the ethnicity variable in relation to what BRÅ in its 2005 report referred to as “factors that may contribute to immigrants committing crimes more often than others” (p. 76).

Overall, attempting to reconstruct the patterns of rapes in Malmö, the following can be inferred from our findings. The typical convicted male rapist is aged 30–40 years, of foreign descent, single and has problems with employment. He somehow knows his victim, who is aged 20–30 years old and in a relationship. He rapes her vaginally while she is under the influence of alcohol and unable to defend herself. The rape takes place in a private realm in the early morning hours, during the weekend and in winter months. These findings provide a clear picture of the typical circumstances of the typical convicted rapes that occurred in the south of Sweden within the past 5 years.
